# SiRNA silencing efficacy prediction based on a deep architecture

**DOI:** 10.1186/s12864-018-5028-8

**Published:** 2018-09-24

**Authors:** Ye Han, Fei He, Yongbing Chen, Yuanning Liu, Helong Yu

**Affiliations:** 1 School of Information Technology, Jilin Agricultural University, Changchun, China; 2 School of Information Science and Technology, Northeast Normal University, Changchun, China; 3 Institute of Computational Biology, Northeast Normal University, Changchun, China; 4 Key Laboratory of Symbolic Computation and Knowledge Engineering of Ministry of Education, Jilin University, Changchun, China; 5 College of Computer Science and Technology, Jilin University, Changchun, China

**Keywords:** siRNA, Deep learning, RNAi

## Abstract

**Background:**

Small interfering RNA (siRNA) can be used to post-transcriptional gene regulation by knocking down targeted genes. In functional genomics, biomedical research and cancer therapeutics, siRNA design is a critical research topic. Various computational algorithms have been developed to select the most effective siRNA, whereas the efficacy prediction accuracy is not so satisfactory. Many existing computational methods are based on feature engineering, which may lead to biased and incomplete features. Deep learning utilizes non-linear mapping operations to detect potential feature pattern and has been considered perform better than existing machine learning method.

**Results:**

In this paper, to further improve the prediction accuracy and facilitate gene functional studies, we developed a new powerful siRNA efficacy predictor based on a deep architecture. First, we extracted hidden feature patterns from two modalities, including sequence context features and thermodynamic property. Then, we constructed a deep architecture to implement the prediction. On the available largest siRNA database, the performance of our proposed method was measured with 0.725 PCC and 0.903 AUC value. The comparative experiment showed that our proposed architecture outperformed several siRNA prediction methods.

**Conclusions:**

The results demonstrate that our deep architecture is stable and efficient to predict siRNA silencing efficacy. The method could help select candidate siRNA for targeted mRNA, and further promote the development of RNA interference.

## Background

In 1988, Fire first introduced RNA interference (RNAi) [1–3], and now it has been found that this mechanism can be detected in many eukaryotic systems, such as mammals, fungi, plants and invertebrates [4]. Small interfering RNA (siRNA) is the production of RNAi, which can induce instant target gene knockdown [3]. RNAi is a vital tool for researching gene function [5–7] and can be used as an effective therapeutic method in the treatment of virus and cancer [8–10].

The gene silencing efficacy of RNAi relies on siRNA design, and many efforts are being made in this area. In early days, several sets of empirical rules to select effective siRNA were proposed according to experimental data. These rules are mainly based on GC content [11], base preferences at specific positions [12, 13], thermodynamic stability [14], internal structure [15] and target mRNA secondary structure [16]. However, these rules are summarized from small scale dataset and can hardly reach our acceptable level. With the accumulation of validated siRNAs, machine learning has been used in effective siRNA recognition. ‘Biopredsi’ is a classical siRNA efficacy prediction method, which are based upon artificial neural network algorithm [17]. Besides, a major siRNA dataset was supplied by Huesken et al. The dataset includes 2431 siRNAs, which were built by high-throughput analysis technology. It has been truly admitted that this dataset is very helpful for the construction of other siRNA efficacy prediction methods [18]. As another artificial neural network, ThermoComposition-21 [19] includes both composition and thermodynamic features. The simple linear method was also used in this area. The method proposed by Jean-Philippe Vert [20] used two kinds of siRNA sequence features as feature set. One is the nucleotides present at each position in the siRNA sequence, the other is the global content of the siRNA in short motifs. It is an accurate and easily interpretable model, and according the experimental results the prediction accuracy of Biopredsi is as accurate as it. Another linear regression model was constructed by nucleotide preference scores [21].

The siRNA efficacy prediction accuracy cannot make us satisfied though the considerable efforts. The reason is the prediction results of most machine algorithms are highly dependent upon the siRNA features, including sequence feature, thermodynamic feature, secondary structure feature, etc. Most of these features are biased and incomplete feature vectors since they are produced by the traditional feature engineering way which is reliant on expert knowledge, and the prediction ability will be limited. Recently, a frontier machine learning algorithm, deep learning, has aroused the attention of researchers. It has been proved that deep learning performed better than the existing machine learning method. Different from the traditional machine learning methods, deep learning framework can conduct the prediction in a data-driven way.

In this paper, we constructed a new siRNA efficacy model based on deep learning algorithm. Firstly, we extracted hidden feature patterns from two modalities, including sequence context features and thermodynamic property. Then we merge them to implement the prediction. For the sequence context features, we utilized convolution layers to automatically learn motif encoding features. In the convolution layers, convolution kernels can be seen as motif detectors, and the potential feature pattern of siRNA multimode motif can be automatically learned by a data-driven method. This method is more abstract and more conductive to prediction and more closely to the essence. The experimental results showed that our deep architecture performed better than the current siRNA efficacy prediction methods in terms of prediction accuracy.

## Methods

### Dataset collection

For siRNA efficacy prediction, we collected 4067 siRNA samples from the dataset of Huesken(2431) [17], Reynolds(248) [12], Vickers (80) [22], Haborth(44) [23], Takayuki(702) [24], Ui-Tei (62) [25] and siRNAdb(500) [26].

In this paper, we divided these siRNA sequences into two datasets by random partition, a training dataset (3660) and a testing dataset (407).

### Encoding of siRNA sequences

There are two encoding method in our paper to transform the siRNA sequences into quantized biological descriptors.

#### Sequence context features

The research which has been reported showed that the sequence context outside the target region effected the efficacy of siRNA [27]. In this paper, the 21 + 2n sequence of n upstream and downstream flanking nucleotides around binding region together with the targeted sites were intercepted.

A siRNA sequence contains 21 bases, including A, U/T, G and C.

1 $$ S={s}_1\cdots {s}_i\cdots {s}_{21},\kern0.5em {s}_i\in \left\{A,U/T,G,C\right\} $$

Then, to perform convolution operation, each sequence contained the flanking region is transformed into a *m*×*k* 2-dimensional matrix. In the matrix, the bases are expressed in four dimensional binary form as follows:

$$ A=<\mathrm{1,0,0,0}>,U/T=<\mathrm{0,1,0,0}>,G=<\mathrm{0,0,1,0}>,C=<\mathrm{0,0,0,1}>. $$

When the length of the flanking region is less than n, the corresponding positions will be encoded to 0.05. The encoding method maps the sequence to a sparse coding and quantifies nucleotides according their relative position.

#### Thermodynamic properties

Some researchers indicate that the efficacy of siRNA relys highly on the thermodynamic stability profile of the siRNA duplex [21]. The guide strand selection mechanism might be reflected due to the differential thermodynamic stability of siRNA duplex [25]. In this section, we select 20 thermodynamic properties as another encoding modality for each siRNA sequence. The details are shown in Table 1. Thermodynamic parameters for the calculations can be found in Ref. [28].

**Table 1:** The thermodynamic properties for siRNA efficacy prediction

Thermodynamic property	Number of features
Stability of hybridization formed between siRNA and mRNA	1
Differential thermodynamic stability of siRNA duplex ends	1
thermodynamic parameter of every two base pairs along the siRNA duplex antisense strand	18

### The deep model construction

**Fig. 1:**
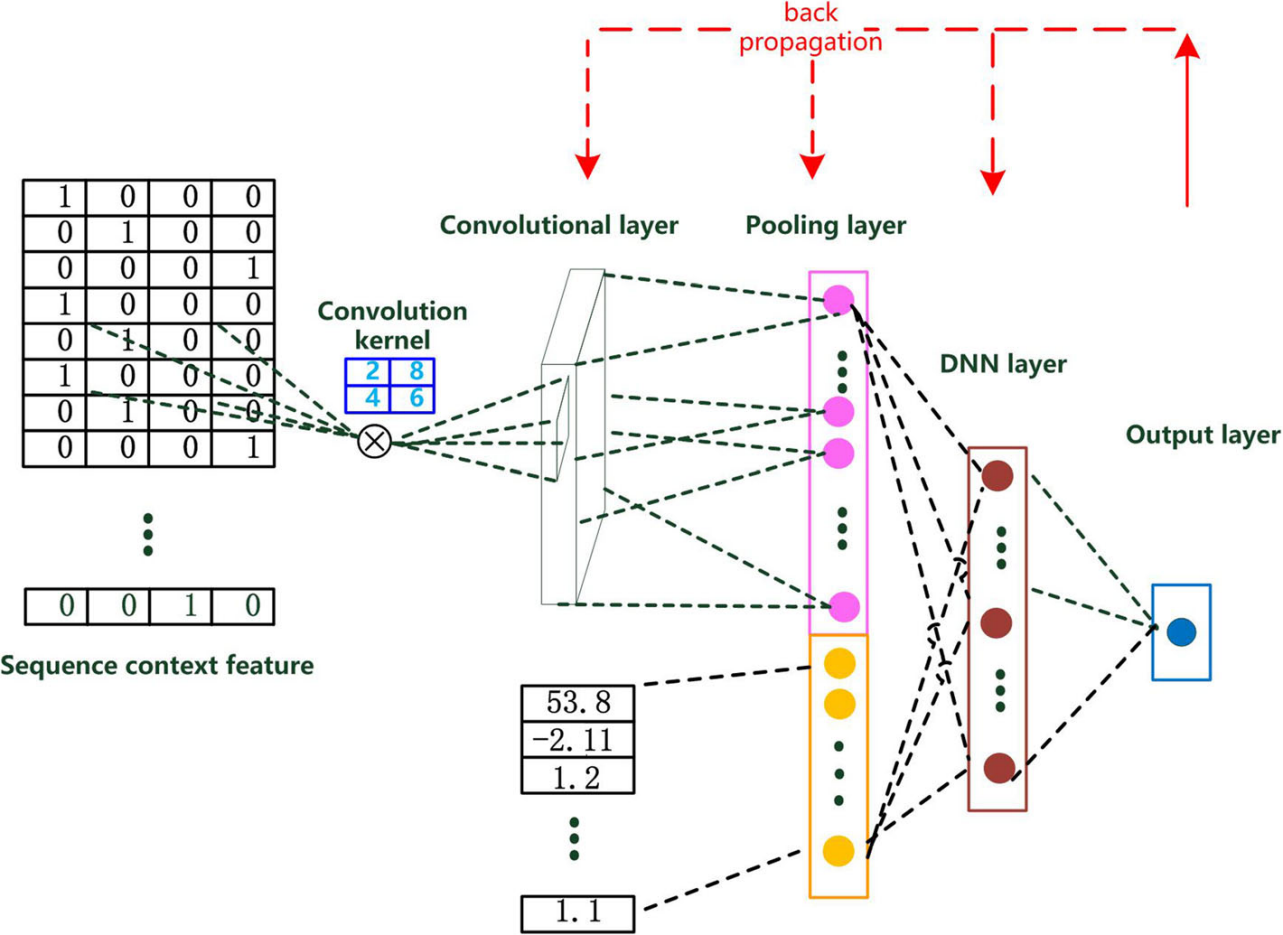
The deep architecture of our siRNA eddicacy prediction model

Figure 1 shows the deep architecture of our siRNA efficacy prediction model. The above mentioned two kinds of features were separately processed and their outputs were merged for efficacy prediction.

In our deep architecture, there are a convolutional layer and a pooling layer in view of the sample size and computational complexity. In the convolutional layer, there are multiple convolution kernels, which have different sizes. These convolution kernels can be seen as motif detectors, which can help us find the motifs playing important role in siRNA efficacy prediction. And through the convolutional operation we can get the corresponding motif encoding features. Most of the existing siRNA efficacy prediction methods encode the sequence according to the experience, but our deep architecture is different from them. The features of our method are trained by siRNA datasets. The feature extraction method has more information, guidance quality and usability. Then the pooling layer can select the most representative motif feature pattern as the feature representation.

The thermodynamic properties and pooling layer are merged into a mixed representation. After batch normalization, we introduce a Deep Neural Network (DNN) to generate their deep representations. Then to produce the efficacy prediction result, 1-state output layer is fully connected to the DNN layer by the logistic regression function. In this paper, we used sigmoid function to perform a linear weighting. Sigmoid function is well adapted for removing the errors aroused by the singular points since the higher gain is in the central area of sigmoid function and the lower gain is in both sides of sigmoid function. Besides, the output values of sigmoid function are in the range of [0,1], which is matched with the range of siRNA efficacy. Therefore, the output efficacy can be calculated as follows:

2 $$ efficacy= sigmoid\left(\sum \limits_{i=1}^n{w}_i{h}_i\right) $$

*h*_*i*_ is the output value of DNN layer, and *w*_*i*_ is the connection weight.

### The design of motif detector

To explore the potential feature pattern included in the siRNA sequence, we designed various convolution kernels. The large-scale training samples are used to correct the weights of convolution kernels by back-propagation algorithm, which guarantee that we can obtain the effective feature pattern.

Firstly, the sequence contained in the flanking region is transformed into (21 + 2*n*) × 4 2-dimensional matrix and every base is expressed as a four dimensional binary code. Besides, the size of convolution kernel is specified as *m* × 4 (2 ≤ *m* ≤ 20). Based on this, we can detect the function of multimode motifs to siRNA efficacy prediction. In this part, the convolution operation is shown as follows:

3 $$ {x}_k=\sum \limits_{j=1}^m\sum \limits_{i=1}^4{\delta}_k{S}_{k+j-1,i}{M}_{j.i} $$

In this formula, *S* represents the sequence of flanking nucleotides around binding region together with the targeted sites and *M* is the *m* × 4 convolution kernel. *x*_*k*_ is the neuron of convolutional layer (1 ≤ *k ≤* 22-*m*), and *δ*_*k*_ is the learning rate for correcting weights. The convolution result is a (22-*m*) × 1 matrix, which represents the feature pattern of every multimode motif.

Secondly, we need an activation function to increase the convolution layer’s nonlinear factors. The experimental result demonstrates that ReLU has good performance. We have given the output *y*_*k*_ below.

4 $$ {y}_k=\max \left(0,{x}_k\right) $$

The purpose of pooling layer is to get the most representative convolution result and get rid of the irrelevant information. In our pooling layer, we carried out average pooling and max pooling to hold the most distinct whole information and local feature representation of the convolution result. Therefore, the pooling result is *y* = (*y*_max_, *y*_*avg*_).

5 $$ {y}_{\mathrm{max}}=\max \left({y}_1,\cdots, {y}_k\right) $$

6 $$ {y}_{avg}= avg\left({y}_1,\cdots, {y}_k\right) $$

Because there are various convolution kernels in the convolution layer, the output of pooling layer is 2*d*-dimensional vector, where d is the number of convolution kernels.

### Assessment of the prediction system

To assess the model efficacy, we adopted two indices, including Pearson Correlation Coefficient (PCC) and the area under the ROC curve (AUC).

PCC is designed to depict the relativity between actural and predicted siRNA efficacies.

7 $$ PCC=\frac{1}{n-1}\sum \limits_{i=1}^n\left(\frac{X_i-\overline{X}}{\sigma_X}\right)\left(\frac{Y_i-\overline{Y}}{\sigma_Y}\right) $$

where *X*_*i*_ and $\overline{X}$ are the actural value and mean value respectively, and *n* is the number of siRNA sequence.

AUC is used extensively to measure the overall performance of prediction model. A higher PCC and AUC indicate the model performs well.

ROC (Receiver Operating Characteristic) curve is generated by plotting sensitivity versus 1-specificity, which is also an indicator to compare the efficiencies of different methods. Sensitivity and specificity are defined as followed:

8 $$ \mathrm{Sensitivity}= TP/\left( TP+ FN\right) $$

9 $$ \mathrm{Specificity}= TN/\left( TN+ FP\right) $$

where *TP* is the number of true positives; *FN* is the number of false negatives; *TN* is the number of true negatives and *FP* is the number of false positives.

## Results and discussion

In this section, we will interpret our experimental results of different parameters. In every experiment, 10-fold cross-validation is conducted to obtain the best parameters.

### The influence of the length of flanking nucleotides on prediction result

**Fig. 2:**
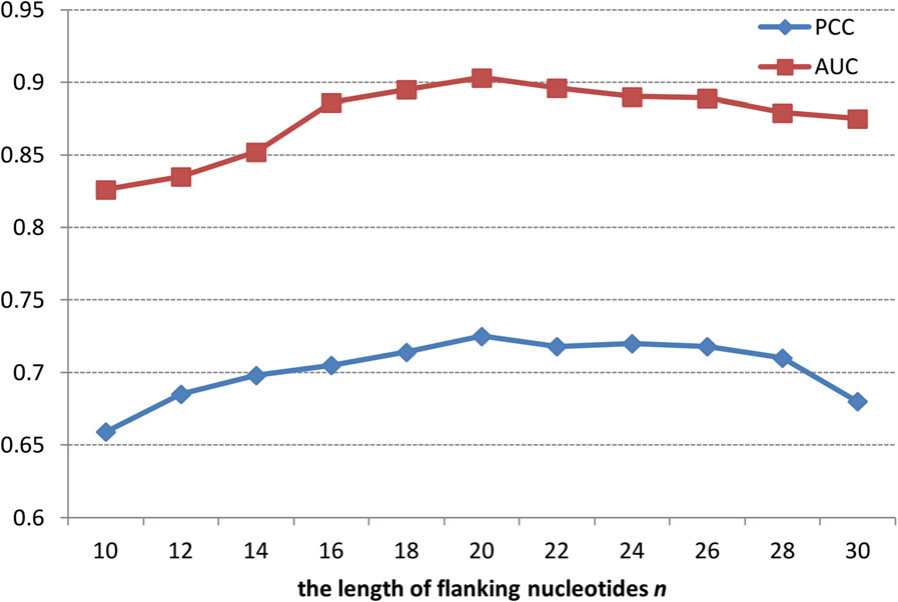
The influence of the length of flanking nucleotides on prediction result

The first parameter is the *n*, which is the length of flanking nucleotides around binding region. The best appropriate *n* to our model should be determined, because it has greater immediate relevance on the prediction results. In this paper, we designed a series of tests using the length of flanking nucleotides *n* from 10 to 30. With regard to each window length, we coded all training siRNA sequences and trained our model. Then, the trained model was designated to predict the input modality of validation sequences. Figure 2 showed the the performance of different *n*.

Figure 2 showed when *n* equals to 20, the prediction result achieves the best performance. The results indicated that our model needs more sequence information to detect more useful deep features.

### The infuence of hyper parameters on prediction result

This part mainly discussed the influence of different hyper parameters on prediction result. In our deep architecture, there are three hyper parameters directly affecting the model’s robustness and deciding the structure of network, including the size of convolution kernel, activation function and learning rate. There comparative experiments were conducted to search the optimal hyper parameters for our deep architecture.

#### The size of convolution kernel

**Fig. 3:**
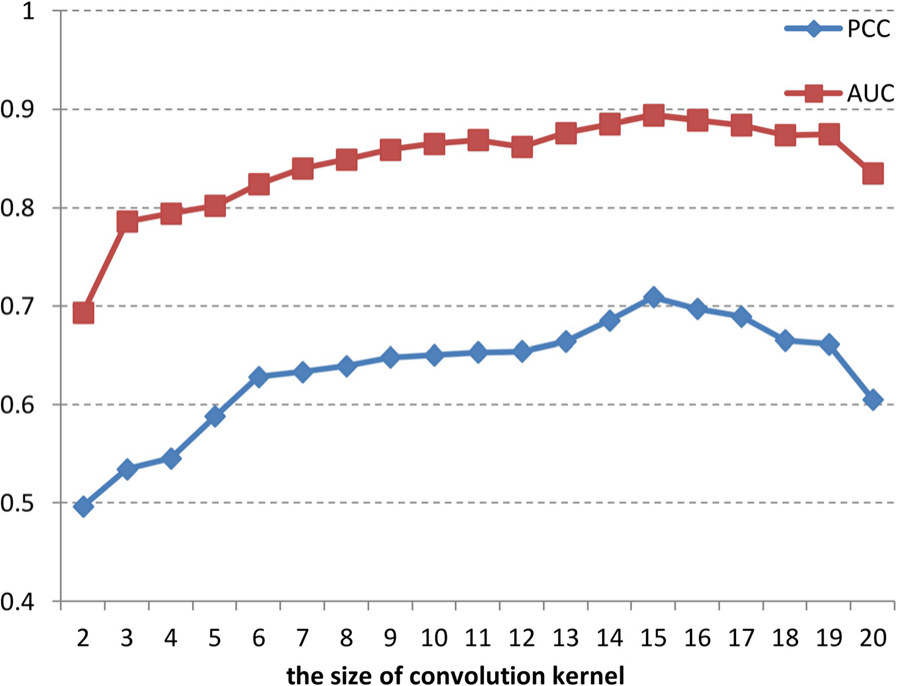
The influence of the size of convolution kernel on prediction result

To learn the feature representation of different multimode motifs, we would like to select the convolution kernels with different sizes. Because there are 21 nucleotides in siRNA sequence, we considered the length of the detected multimode motifs can be defined as the value less than 20 and larger than 2. Consequently, we employed 19 *m* × 4 convolution kernels to learn the feature of multimode motif. To get the most appropriate hyper parameter, we constructed 19 deep neural networks. In every network, the value of *m* is different and the corresponding number of convolution kernel is 22-*m*. The performance of different *m* can be observed in Fig. 3.

As shown in Fig. 3, different convolution kernels influence the prediction results. When *m* equals to 15, the prediction result achieves the best performance. The result indicates that the convolution kernels we designed could learn the effective feature pattern from the input modality. Next, we analyze the effect of *m* on prediction result. Figure 3 shows that when m is increasing, the prediction result of corresponding deep neural network becomes larger, but when m is larger than 15, the prediction result lower. We speculate that the reason could be the convolution kernels with smaller size only discover the information associated with low-mode motif and neglect the contribution of high-mode motif and the whole sequence feature. And when the size of convolution kernel is becoming increasing, the feature representation of high-mode motif will be detected and the contribution of low-mode motif will be neglected. The result in either case can give rise to the decrease of prediction result. Therefore, we should choose the reasonable size of convolution kernel to achieve effective motif feature learning. In this paper, we designed a convolution kernel set, and PCC of the convolution kernels contained in the set are higher than 0.6, which guarantee the learning feature pattern has adequate discriminating ability.

#### Activation function

**Fig. 4:**
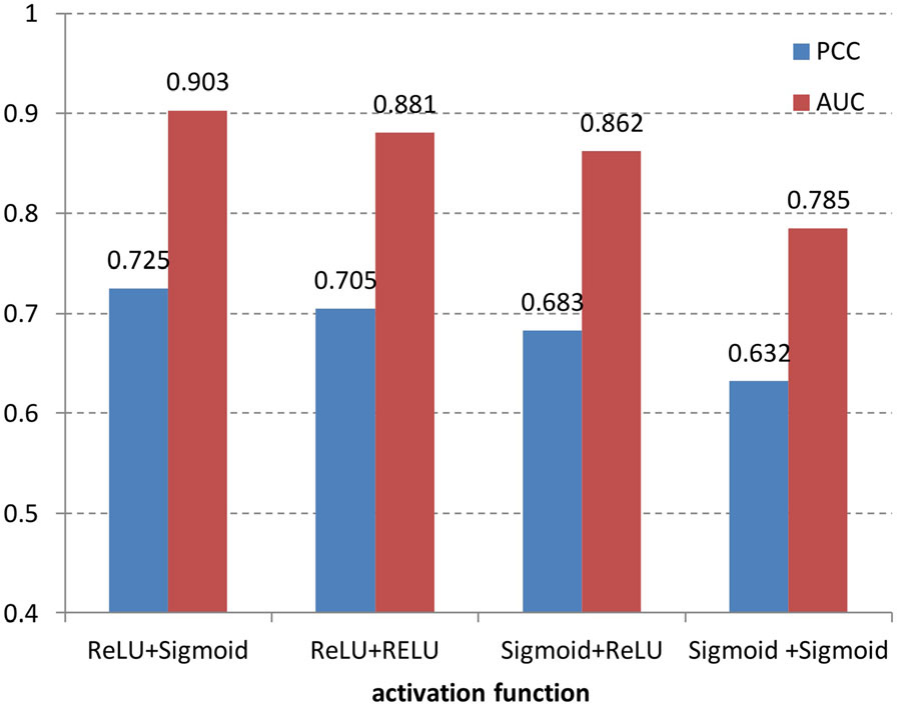
The influence of activation function on prediction result

As different activation functions influence the distinguish ability and rationality of incoming signal mapping to feature space, we next construct four deep models by utilizing different activation functions and compare the prediction results. Our deep architecture needs two activation functions, one is in convolution layer and the other is in the DNN layer. Sigmoid, tanh and RELU are three common activation functions. But tanh is not suitable for detecting the local tiny features of motif since it is often used in the condition that features have big difference. The performances of RELU and sigmoid function in the convolution layer and DNN layer are shown in Fig. 4.

From Fig. 4 we can see that the prediction results with different combinations of activation function are diverse and the first combination performs best (ReLU + Sigmoid). The result shows that in the convolution layer the better choice is ReLU function. The reason may be that sparsity is added into the output feature of convolution layer by ReLU, and this way can enhance the nonzero neurons’ information. In the DNN layer, sigmoid can be used as activation function because it can summarize the contribution of all feature representation. Besides, output of sigmoid is from 0 to 1, which is consistent with the range of siRNA efficacy.

#### Learning rate

**Fig. 5:**
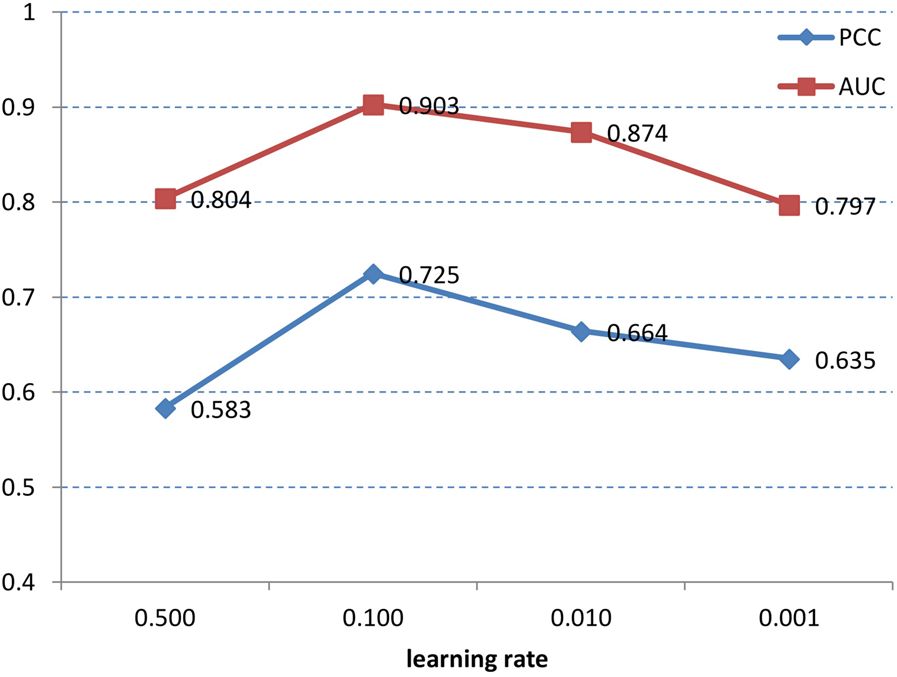
The influence of learning rate on prediction result

Learning rate can control the speed of weight correcting, and a suitable learning rate can obtain the best weights of neural network. However, learning rate cannot be decided by experience but experiment. Too large learning rate will make neural network lose the optimal weights and sink into relative extremum, and too small learning rate will bring about slow convergence speed and insensitivity to error correction. In this paper, we chose diverse learning rates, 0.5, 0.1, 0.01 and 0.001, to carry out comparative the experiments. The termination condition is that the iteration time is more than 1000 or error is smaller than 0.001. Figure 5 shows the impact of learning rate on prediction results.

Figure 5 shows that when the learning rate equals to 0.1, we can get the best result. Besides, it can be found that when the learning rate equals to 0.5, the result is lowest. It shows that the neural network has lost the optimal weights and sank into relative extremum. Then when the learning rate equals to the other two values, PCC and AUC are relatively low. The reason may be that when the iterative is 1000 the network has slower convergence speed and cannot get the best weight. According to the prediction accuracy and training time, our deep architecture set the learning rate to 0.1.

### Compared with other algorithms

From what has discussed above, our deep architecture has 15 convolution kernels with the size from 6 × 4 to 20 × 4. Through the convolution operation, we got 15 feature maps with size (22-m) × 1, each of which then was processed by max pooling and average pooling with (22-m) pooling size respectively in the following pooling stage. Thus, after such pooling operation, each input was transformed into 2 × 15 × 1 vector. The 15 kernels with different size are transformed into a 30-dimensional vector in the pooling layer. The activate function of convolutional layer is ReLU, the activation function of DNN layer is sigmoid, and the learning rate is 0.1. There are 25 nerouns in DNN layer.

**Fig. 6:**
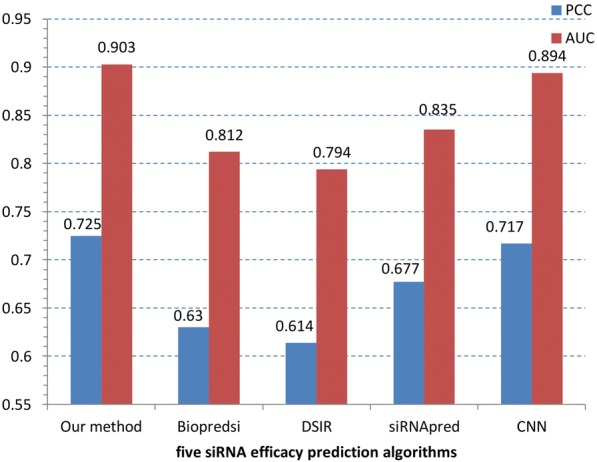
The comparison among five algorithms

Furthermore, we compared our deep neural network with some siRNA efficacy prediction methods, including siRNApred [29], Biopredsi [17], DSIR [20] and CNN [30]. The prediction results of the five methods are shown in Fig. 6.

From Fig. 6, it can be found out that our deep architecture performed best, reaching at 0.725 PCC and 0.903 AUC.

The most probable reason is that Biopredsi, DSIR and siRNApred are the traditional machine learning methods, which belong to the feature engineering way and rely on expert knowledge. And our deep learning methods can supply non-linear mapping operations and multiple layer networks to detect potential complex patterns and generate homogenous deep representations for prediction tasks. Therefore the performance of Biopredsi, DSIR and siRNApred are less than our deep architecture.

And we can find that the performance of our method is better than CNN. The method CNN used the feature of siRNA sequence and developed a convolutional neural network including a convolution layer and a pooling layer. Because the sequence cannot fully reflect the siRNA properties and the efficacy of siRNA strongly depends on the thermodynamic stability profile of the siRNA duplex, we used siRNA context feature and thermodynamic properties and added a DNN layer to combine the two types of feature. Because their components depict the feature of siRNA from different points of view, the fully connected DNN structure could interconnect all factors for their joint effect in its hidden states.

## Conclusions

As a common molecular tool, siRNA can research gene function and be used as an effective therapeutic method in the treatment disease. Numerous methods have been developed to design active siRNA. However, the siRNA efficacy prediction accuracy cannot make us satisfied. In this study, we proposed a new siRNA efficacy prediction method based on a deep architecture. Comparing with the existing method Biopredsi, DSIR, siRNApred and CNN, our method performs best. The results show that our deep architecture could tap the contribution of siRNA context sequence and thermodynamic properties on efficacy prediction. Besides, our method can extract the valuable information contained in the feature pattern. Finally, the data-driven feature learning pattern outweighs the learning pattern which mainly depends on the expert knowledge.
